# Improvement Activity of 1-Deoxynojirimycin in the Growth of Dairy Goat Primary Mammary Epithelial Cell through Upregulating LEF-1 Expression

**DOI:** 10.1155/2018/7809512

**Published:** 2018-02-19

**Authors:** Shengyue Ji, Ming Liu, Yuping Zhang, Hongfu Zhang

**Affiliations:** ^1^Key Laboratory of Animal Nutrition, Institute of Animal Sciences, Chinese Academy of Agricultural Sciences, Beijing 100193, China; ^2^College of Animal Science and Technology, Hunan Agricultural University, Changsha 410128, China; ^3^Precision Livestock and Nutrition Unit, Department of AgroBioChem, Université de Liège, Passage des Deportes 2, 5030 Gembloux, Belgium

## Abstract

LEF-1/wnt10b is one of the most important signaling pathways regulating mammary gland growth and development and is also a potential target for molecular breeding. In this work, 1-deoxynojirimycin (DNJ), a natural alkaloid extracted from plant mulberry or microorganism, was found to have a positive activity in primary breast epithelial cell growth of dairy goats. The findings showed that, compared to the control, 6 *μ*M DNJ in the DMEM/F12 medium* in vitro *greatly improved the density of dairy goat breast epithelial cell and significantly increased the LEF-1 mRNA level (*P* < 0.01) and thus enhanced cell growth. In addition, DNJ displayed a similar function in alleviating the growth suppression of epithelial cell and the decrease of LEF-1 mRNA level resulting from lentiviral-mediated LEF-1 knockdown. Simultaneously, no significant change of the mRNA levels of IGF-1 and Fgf10, the other two key regulators in mammary gland growth and development, could be detected. Furthermore, the mammary duct of DNJ-fed mouse illustrated a better development accompanied with a higher LET-1 mRNA level than that of the control. In conclusion, DNJ could improve breast epithelial cell growth through upregulating LEF-1 expression, which supplied a new means in studying mammary gland growth and development.

## 1. Introduction

Mammary gland growth and development is one of the key events belonging to animal reproductive performance, which is the result of the activities of major genes or related signaling pathways, such as wingless signaling (Wnt), fibroblast growth factor (FGF), and insulin-like growth factor (IGF) pathway [1]. LEF-1, a member of the T-cell specific factor (TCF) family, is a key regulator of Wnt signaling pathway. It mediates *β*-catenin dependent transcription factors that bound to the specific promoters with conserved sequence CTTTGT [2] and plays a critical role in the canonical Wnt signaling pathway in early mammary gland morphogenesis.

The mammary gland growth and development of LEF-1 KO mice is abnormal: the two pairs of placodes are deficient, and other formed pairs are tiny and degenerated subsequently [3, 4]. And the depletion of LEF-1 results in downregulation of cyclin D (CycD) and robustness of matrix metallopeptidase 7 (MMP-7) [5, 6]. In recent years, LEF-1 had been identified as a potential target for molecular breeding and thus had attracted more and more attention from related researchers.

1-Deoxynojirimycin (DNJ), a natural alkaloid from plant mulberry or microorganism, is a conventional hypoglycemic drug in clinical application for effectively inhibiting *α*-glucosidase activity [7, 8]. Thus, it could potently inhibit the metabolism of saccharides and lower blood glucose levels. Besides, DNJ sourced from* Bacillus subtilis* could significantly increase the levels of adiponectin (that is efficient in lowering blood glucose levels and increasing insulin sensitivity) and its receptor in differentiated 3T3-L1 adipocytes [9].

Recently, other important functions of DNJ were also studied. It has been investigated as an agonist of natural killer (NK) cell receptors and accordingly displayed a remarkable activity towards the receptors of both NKR-P1A (rat) and CD69 (human) [10]. In addition, DNJ also showed an ability in attenuating high glucose-accelerated senescence in human umbilical vein endothelial cells [11]. Moreover, DNJ could inhibit HUVEC proliferation by inducing G1 phase cell cycle arrest and apoptosis showing a somatostatin mimetic effect [12].

In the present study, 6 *μ*M DNJ in DMEM/F12 medium could increase the density of dairy goat mammary epithelial cell* in vitro* accompanied with a significant improvement of LEF-1 mRNA level. Besides, both the suppression of epithelial cell growth and the decrease of LEF-1 mRNA level elicited by lentiviral-mediated LEF-1 knockdown vector were all attenuated by DNJ. In addition, the feeding experiment revealed that DNJ could enhance mammary duct development* in vivo*. These findings suggested that DNJ could accelerate the growth of primary mammary epithelial cell through upregulating LEF-1 expression, which provided a specific means of studying mammary gland growth and development.

## 2. Materials and Methods

### 2.1. Vector Preparation

The lentivirus-mediated LEF-1 knockdown vector (Lenti-LEF-1) and the adenovirus-mediated LEF-1 overexpression vector (pAd-LEF-1) were obtained from the Fox Chase Cancer Center (Philadelphia, USA). They were packaged in HEK293T/A, respectively, to construct infectious viral vectors for infecting the primary cells.

### 2.2. Cell Isolation and Culture

Using collagenase-I/hyaluronidase digestion mothed according to the reports [13, 14], four 240-day Saanen Dairy Goats with similar bodyweight were sacrificed to isolate primary mammary epithelial cell. The primary mammary epithelial cells were seeded into a 6-well plate at the density of 3 × 10^5^/cm^2^. Subsequently the cells were treated under DNJ solution with different concentration for 72 h and then observed under a microscope (100x). All animal experiments in this work were approved by the Animal Ethical Committee of the Chinese Academy of Agricultural Sciences (Beijing, China).

### 2.3. RNA Extraction and qRT-PCR

Total RNA in primary mammary epithelial cells was isolated using TRIzol reagent (Invitrogen) following the manufacturer's instructions. The resultant RNA was digested by Rnase-free Dnase-I (Fermentas) to shield genomic DNA contamination according to the manufacturer's manual. Subsequently real-time qPCR was used to evaluate the gene expression profiles. Real-time qPCR reactions were carried out in a final volume of 25 *μ*l, which contained SYBR Premix Ex Taq (TaKaRa), 0.4 mM of each primer, and 200 ng of cDNA template. Each individual sample was run in triplicate wells. PCR amplification cycles were performed in iQ™5 Multicolor Real-Time PCR Detection System (Bio-Rad), using SYBR Premix Ex Taq II kit (TaKaRa). The reactions were initially denatured at 95°C for 3 min followed by 40 cycles of 95°C for 15 s, 60°C for 20 s, and 72°C for 30 s. The melting curve analysis was carried out after amplification to verify the accuracy of each amplicon. And the density of SYBR green I and the threshold cycle (Ct) value [15] were determined by using iQ5 Optical System Software 2.1.

### 2.4. Western Blotting

Each protein sample of 25 *μ*g was separated by using 12% SDS-PAGE and subsequently was electrotransferred to PVDF membrane (Millipore) for immunoblot analysis. The following primary antibodies were used: anti-LEF-1 (Kamiya, MC-865, 1 : 300) and anti-*β*-actin (Abbkine, A01010, 1 : 800) which was applied as the loading control. After incubation with the appropriate HRP-conjugate secondary antibody, proteins were detected by using a ChemiDoc XRS imaging system and analysis software Quantity One (Bio-Rad).

### 2.5. DNJ Feeding Experiment and H&E Staining

Experiments were conducted using 12 Chinese Kunming mice (Kunming Institute of Zoology, China), weighing 20 ± 2 g. Animals were housed in stainless steel cages by gender in a ventilated animal room with temperature and relative humidity of 22 ± 2°C and 55 ± 10%, respectively, in a 12 h light/dark cycle for 7 days prior to treatments. Sterilized food and distilled water for mice were available ad libitum. These mice were in average divided into 2 groups, one of which was fed with normal diet as control group, and the other was fed with normal diet added with DNJ at 0.5 *μ*g/g bodyweight. After 2 months, the mammary gland of these mice was excised and applied to manufacture cryosections. Subsequently the cryosections were stained with H&E (hematoxylin-eosin) and observed under a microscope (40x).

### 2.6. Statistical Analysis

All data were expressed as mean ± SE and were analyzed by ANOVA with Dunnett's test after normalization. The differences were considered to be significant at *P* < 0.05 and extremely significant at *P* < 0.01.

## 3. Results

### 3.1. Improvement Activity of DNJ in Dairy Goat Primary Mammary Epithelial Cell Growth and LEF-1 mRNA Level

**Figure 1:**
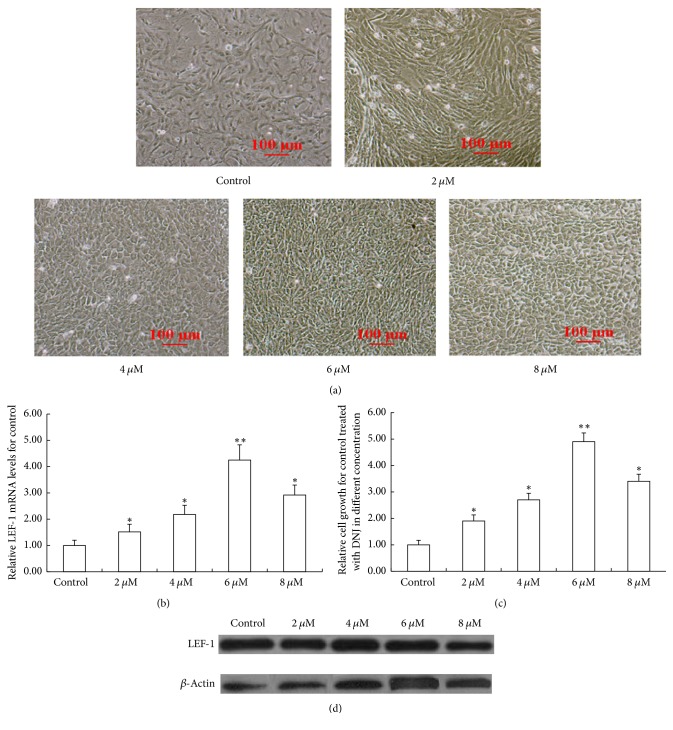
Effect of DNJ on the growth of dairy goat primary mammary epithelial cells. (a) Effect of a series of DNJ solutions with different concentration on the primary mammary epithelial cell growth. Dairy goat primary mammary epithelial cells were seeded into a 6-well plate at the density of 3 × 10^5^/cm^2^. Then the cells were treated with a series of DNJ solutions with different concentration for 72 h and were observed under a microscope (100x). (b) LEF-1 mRNA levels in the primary mammary epithelial cells treated with a series of DNJ solutions with different concentration were detected by using real-time qPCR. (c) Relative growth of the primary mammary epithelial cell in each group of (a). (d) The protein levels of LEF-1 in mouse mammary glands were detected using western blotting. Data were expressed as mean ± SEM, compared with control (DMSO); *∗* indicated *P* < 0.05; *∗∗* indicated *P* < 0.01.

The primary mammary epithelial cells were seeded into a 6-well plate at the density of 3 × 10^5^/cm^2^, and the wells were added with DNEM/F12 medium containing 2 *μ*M, 4 *μ*M, 6 *μ*M, and 8 *μ*M DNJ, respectively. After 72 h, the densities of primary mammary epithelial cells increased in different levels, and the most remarkable increase of cell density presented in the 6 *μ*M DNJ group (Figures 1(a) and 1(c)). Also LEF-1 mRNA and protein levels in each DNJ-treatment cell were detected individually. And they arose more or less among the different groups (Figures 1(b) and 1(d)), and the increase degrees were consistent with those of cell densities in different levels, and the optimal DNJ concentration for increasing LEF-1 mRNA and protein level was 6 *μ*M that was used in the following experiments.

### 3.2. DNJ Enhanced Mammary Epithelial Cell Growth via Upregulating LEF-1 Expression

**Figure 2:**
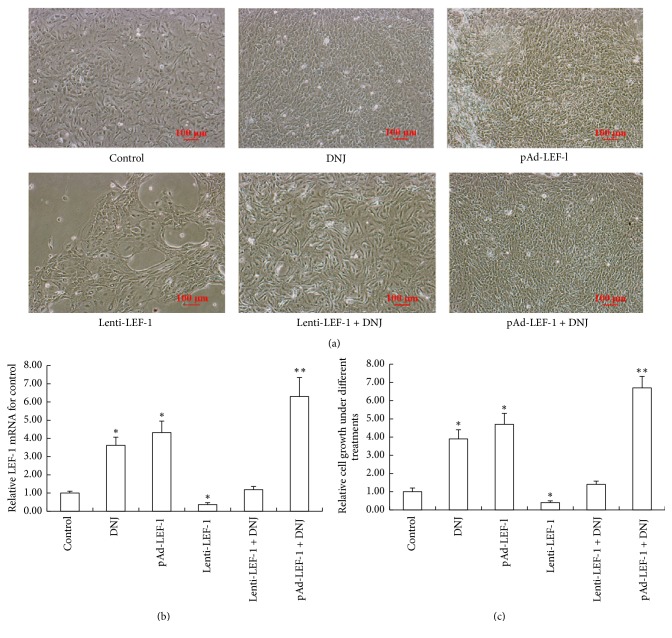
Improvement activity of DNJ in primary mammary epithelial cell growth through upregulating LEF-1 expression. (a) A similar effect of DNJ with LEF-1 overexpression on the primary mammary epithelial cell growth. Dairy goat primary mammary epithelial cells were added to a 6-well plate at a density of 3 × 10^5^/cm^2^, which were treated with 6 *μ*M DNJ, adenovirus-mediated LEF-1 overexpression vector (pAd-LEF-1), lentivirus-mediated LEF-1 knockdown vector (Lenti-LEF-1), Lenti-LEF-1 + 6 *μ*M DNJ, or pAd-LEF-1 + 6 *μ*M DNJ for 72 h, respectively, and observed using a microscope (100x). (b) Detection of LEF-1 mRNA levels in each group of (a) through real-time qPCR. (c) Relative growth of the primary mammary epithelial cell in each group of (a). Data were expressed as mean ± SEM, compared with control (DMSO); *∗* indicated *P* < 0.05; *∗∗* indicated *P* < 0.01.

The result illustrated that DNJ showed a promotion effect on improving the primary mammary epithelial cell growth and reducing cell growth inhibition induced by lentivirus-mediated LEF-1 knockdown vector (Figures 2(a) and 2(c)). At the same time, DNJ treatment supplied pAD-LEF-1-mediated LEF-1 overexpression vector that resulted in LEF-1 overexpression that could synergistically display a remarkable effect on increasing the primary mammary epithelial cell density (Figures 2(a) and 2(c)). Besides, the LEF-1 mRNA level alternation in different groups was correspondingly consistent with that of the cell density changes (Figures 2(a), 2(b), and 2(c)). These findings suggested that DNJ enhanced mammary epithelial cell growth via upregulating LEF-1 expression.

### 3.3. DNJ Exerted an Improvement Activity via Wnt but Not FGF or IGF Signaling Pathway

**Figure 3:**
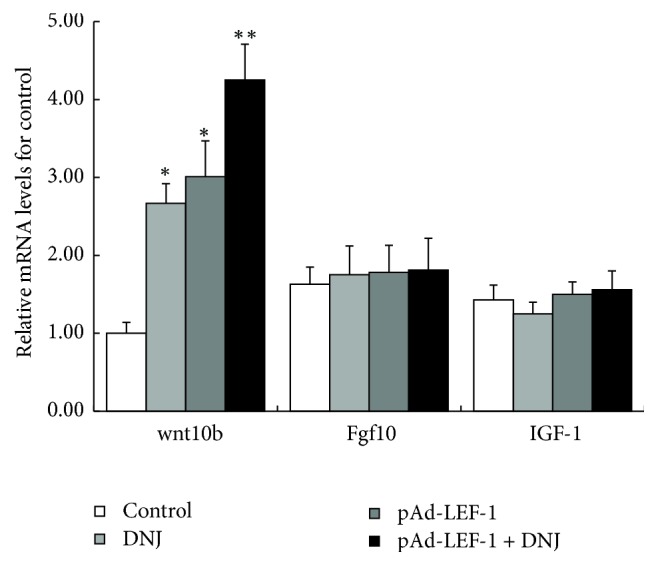
DNJ exerted improvement activity via Wnt but not FGF or IGF signaling pathway. The levels of Wnt10b, Fgf10, and IGF as marker gene in Wnt, FGF, and IGF signaling pathways were detected, respectively, using real-time qPCR, from the cells treated with 6 *μ*M DNJ, pAd-LEF-1, or pAd-LEF-1 + 6 *μ*M DNJ for 72 h, respectively. *∗* suggests that the difference between the treatment and the control is statistically significant (*P* < 0.05), and *∗∗* suggests that the difference between the treatment and the control is very statistically significant (*P* < 0.01).

The mRNA levels of the marker genes in Wnt, FGF, and IGF signaling pathway, Wnt10b, Fgf10, and IGF-1, in the cells treated with 6 *μ*M DNJ, pAd-LEF-1, or both for 72 h, were detected by using real-time qPCR. The results showed that only Wnt10b mRNA level raised response to the treatments, while the mRNA levels of Fgf10 or IGF-1 had no significant change under the treatments (Figure 3). These findings suggested that DNJ improved the growth of primary mammary epithelial cells through upregulating signaling pathways of LEF-1/Wnt10b, but not through FGF or IGF signaling pathway.

### 3.4. Improvement Activity of DNJ in Mammary Duct Development* In Vivo*

**Figure 4:**
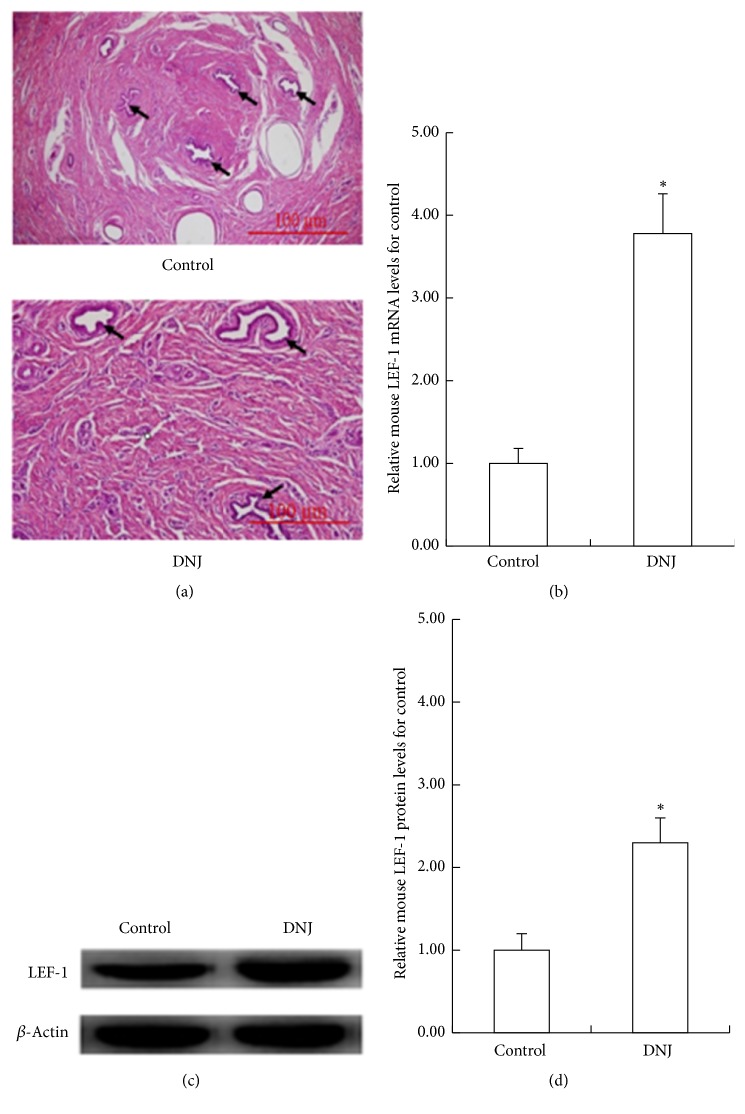
Improvement activity of DNJ in mammary duct development in vivo. (a) H&E staining cryosections of mammary ducts of mouse fed with diet supplied with DNJ or not, which were observed under a microscope (40x). The arrows indicated the mammary ducts. mRNA levels (b) and protein levels ((c), (d)) of LEF-1 in mouse mammary glands were detected, respectively, using real-time qPCR and western blotting. *∗* suggests that the difference between the treatment and the control is statistically significant (*P* < 0.05), and *∗∗* suggests that the difference between the treatment and the control is very statistically significant (*P* < 0.01).

2 groups of mice were, respectively, fed with diet with added DNJ or not. After 2 months, their mammary glands were excised and cryosections were formed with H&E staining. The results showed that, observed under a microscope (40x), mammary ducts from DNJ-treated group appeared more bulky than that from the control group (Figure 4(a)). At the same time, the levels of LEF-1 mRNA and its protein in mammary glands in DNJ-treated mouse were significantly higher than that of the control (Figures 4(b), 4(c), and 4(d)). These findings confirmed the above result again that DNJ could improve mammary epithelial cell growth and upregulate LEF-1 expression.

## 4. Discussion

In recent years, DNJ has been attracting more and more attention for its widespread clinical application not only in decreasing blood glucose but also in treating other diseases [16, 17]. In this current work, DNJ displayed a specific function in improving the growth of primary mammary epithelial cells of dairy goats. And 6 *μ*M DNJ in DMEM/F12 medium could greatly increase the density of dairy goat mammary epithelial cell* in vitro*. It is documented that DNJ with a concentration of up to 5 *μ*M had no toxicity to differentiated 3T3-L1 adipocytes [9]. The findings in this study demonstrated that dairy goat primary mammary epithelial cells had a higher tolerance than that document reported.

The development of mammary tissue mainly consisted of the formation and specialization of dairy line, formation of mammary placode, glandular bud, and primitive ductal branching [18–20]. And this process was tightly and complicatedly regulated by several signaling pathways including Wnt, FGF, and IGF. In the Wnt signaling pathway, Wnt 10b and the Wnt reporter (TOP-Gal) played key roles in mammary line formation and specialization. And Fgf10 was expressed at first in the formative stage of mammary line and regulated FGFR2b to influence Wnt10 response to mammary line development [21]. In addition, FGF10^−/−^ and FGFR2b^−/−^ mice displayed developmental defects of mammary bud [22]. IGF signaling pathway was essential for mammary development, and IGF-I^−/−^ null female animals were reported to have significantly less mammary development than age matched wild-type controls [23].

In Wnt signaling pathway, LEF-1 gene played a role as downstream transcriptional mediator, which controlled the development of mammary through regulating Wnt signaling activity. In this current work, the results showed that DNJ could alleviate the growth suppression of the epithelial cells and the decrease of LEF-1 mRNA level elicited by lentiviral-mediated LEF-1 knockdown. However, Fgf10 and IGF-1, another two key regulators in mammary gland growth and development [24, 25], mRNA levels did not show any change under DNJ treatment, which indicated that DNJ could promote the growth of primary mammary epithelial cells through upregulating LEF-1/Wnt10b but not FGF or IGF signaling pathways. Collectively, LEF-1 gene had a significant role in mammary development, which was important for DNJ in improving mammary epithelial cell growth through enhancing the expression of LEF-1 gene.

## 5. Conclusions

In a summary, DNJ displayed a positive activity in primary mammary epithelial cell growth of dairy goats via upregulating LEF-1 expression. At first, it could* in vitro *greatly improve mammary epithelial cell density and significantly increased the LEF-1 mRNA level (*P* < 0.01), which suggested that DNJ probably enhanced dairy goat mammary epithelial cell growth through upregulating LEF-1 mRNA level. In addition, DNJ displayed a similar function in alleviating the growth suppression of epithelial cell and the decrease of LEF-1 mRNA level elicited by lentiviral-mediated LEF-1 knockdown. Furthermore, the mammary duct of DNJ-fed mouse illustrated a better development than that of control. Thus, DNJ could improve mammary epithelial cell growth through upregulating LEF-1 expression, which supplied a new means of studying mammary gland growth and development.
